# Cardioembolic Stroke Three Days Post-Video-Assisted Thoracoscopic AtriClip in a Patient With Paroxysmal Atrial Fibrillation

**DOI:** 10.7759/cureus.64459

**Published:** 2024-07-13

**Authors:** Sammudeen Ibrahim, Kwasi Asamoah Opare-Addo, Ibrahim Shamasneh, Basilio Addo

**Affiliations:** 1 Internal Medicine, Piedmont Athens Regional Medical Center, Athens, USA

**Keywords:** acute cardioembolic stroke, complications of anticoagulation, left atrial appendage occlusion, atrial fibrillation (af), atriclip

## Abstract

Atrial fibrillation is a global epidemic due to aging and chronic diseases. Treatment options are expanding to prevent thromboembolism in anticoagulant-ineligible patients. The left atrial appendage, implicated in 90% of embolic strokes, is increasingly managed with occlusion devices like the AtriClip.

A 62-year-old woman with prior stroke, severe gastrointestinal bleeding on anticoagulation, and paroxysmal atrial fibrillation experienced sudden left-sided weakness and altered mental status three days post-AtriClip procedure. Brain MRI revealed acute infarcts in the right cerebellum and scattered punctate infarcts in both cerebral hemispheres. No further invasive investigations or interventions were recommended, as they would not influence management. Left-sided weakness improved, and the patient was discharged to a subacute rehabilitation center.

Despite the AtriClip's ability to lower stroke incidence by occluding the left atrial appendage, there remains a residual risk of cerebrovascular events that can significantly impact morbidity and mortality. This case underscores persistent risks despite complete appendage closure, emphasizing the need for broader studies on post-AtriClip stroke risk.

## Introduction

Atrial fibrillation (AF) is widespread, affecting approximately one in three to five individuals over the age of 45. From 2010 to 2019, there has been a notable increase in the global prevalence of AF, with numbers rising from 33.5 million to 59 million individuals [1]. It is projected that by 2050, approximately 15.9 million people will have AF in the United States of America [2]. Evidently, AF is associated with significant cardiovascular outcomes, including cerebrovascular accidents (CVA), resulting in increased long-term disability and mortality [3,4].

The Framingham Heart Study (1998) found a significant 50-90% increase in mortality risk associated with AF, underscoring its global health impact and the imperative for improved strategies. Anticoagulation remains pivotal in reducing cerebrovascular risk for AF patients with congestive heart failure, hypertension, age ≥75, diabetes, stroke, vascular disease, age between 65-74, and female sex (CHADS-VASc) scores > 1 in males and > 2 in females [5]. However, about 10% of patients with AF cannot safely use anticoagulation due to absolute contraindications, such as the life-threatening bleeding associated with its use [6]. These highlighted unmet needs have led to the exploration of other therapeutic options to prevent thromboembolism in patients who are poor candidates for anticoagulation.

The left atrial appendage (LAA) has long been identified as the source of thrombi in up to 90% of ischemic strokes [7]. In recent years, the occlusion of the LAA with devices like the AtriClip and Watchman has become a significant treatment modality aimed at preventing the formation of mural thrombus. Patients at significant risk of stroke, particularly those with contraindications to anticoagulation, stand to benefit from these strategies [8]. However, the AtriClip procedure does not require continuation of anticoagulation as there is no contact with the patient's blood in contrast to the Watchman procedure, which requires a short period of anticoagulation to avoid device-related thrombosis, hence the increased utility of AtriClip in patients with absolute contraindication to both anticoagulation and antiplatelet therapy [8]. 

We present a case of a 62-year-old female with a history of life-threatening bleeding while on apixaban and paroxysmal atrial fibrillation who developed an embolic stroke three days post-AtriClip procedure, along with a literature review on the incidence of stroke following the AtriClip procedure.

## Case presentation

A 62-year-old female with a history of recent cerebrovascular accident (CVA) and paroxysmal atrial fibrillation (PAF), CHADS-VASc score of 6, and a life-threatening lower gastrointestinal bleed while on apixaban for PAF underwent a video-assisted thoracoscopic (VAT) left atrial appendage clip procedure for management of PAF on account of an absolute contraindication to anticoagulation three days prior to presenting with acute onset left-sided weakness and altered mental status. Her last well-known time was approximately eight hours prior to the presentation. In the emergency department, her blood pressure was elevated at 170/86 mmHg; otherwise, her vital signs were stable. On examination, she had a regular pulse, with first and second heart sounds present but no murmurs. The chest was clear to auscultation, and neurological exams revealed power of 2/5 in the left upper and lower extremities, with 2+ reflexes in all extremities and an absent Babinski reflex bilaterally; all other neurological exams were unremarkable.

**Figure 1:**
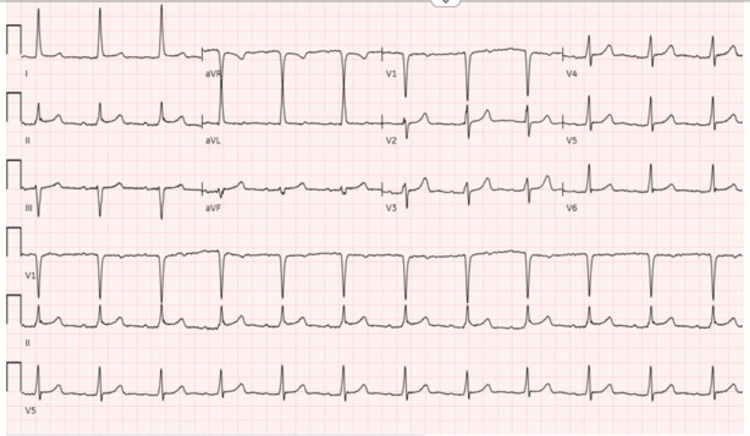
An EKG showed subtle S-T segment elevation in lead II without any reciprocal changes. EKG: electrocardiogram

**Figure 2:**
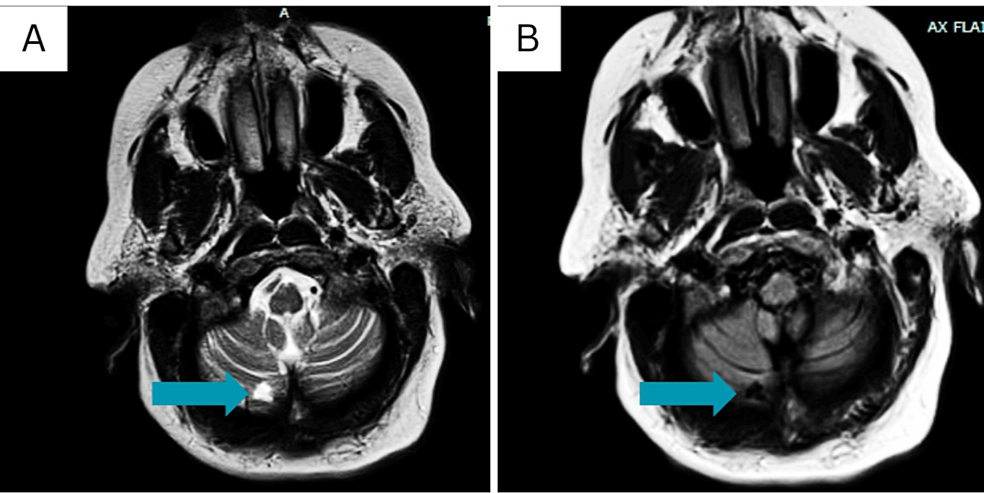
The MRI T2 (A) and FLAIR (B) show an acute right tiny cerebellar infarct (blue arrow). MRI: magnetic resonance imaging; T2: transverse relaxation time; FLAIR: fluid-attenuated inversion recovery

Initial laboratory investigations showed a white blood count of 13.7 x 10^3^/ul, hemoglobin of 10.9 g/dl, and platelets of 338 x 10^3^/ul. High-sensitivity troponins at zero, two, and six hours were 1037 ng/l, 1260 ng/l, and 1222 ng/l, respectively. An electrocardiograph (EKG) showed subtle S-T segment elevation in lead II without any reciprocal changes (Figure 1), and a repeat transthoracic echocardiogram revealed no regional wall abnormalities or mural thrombus. Elevated troponin was thought to be likely due to demand ischemia from the recent AtriClip procedure with subsequent myocardial damage to the left atrial appendage. A prior echocardiogram with a bubble study had shown no evidence of a patent foramen ovale. A computer tomography (CT) angiogram of the head and neck demonstrated approximately 40% stenosis of the proximal internal carotid arteries bilaterally but no acute intracranial abnormality. Magnectic resonance imaging (MRI) of the brain without contrast revealed acute tiny infarcts in the right cerebellum and punctate infarcts scattered in the white matter of both hemispheres, diagnosing acute embolic stroke with multiple infarcts (Figure 2).

**Figure 3:**
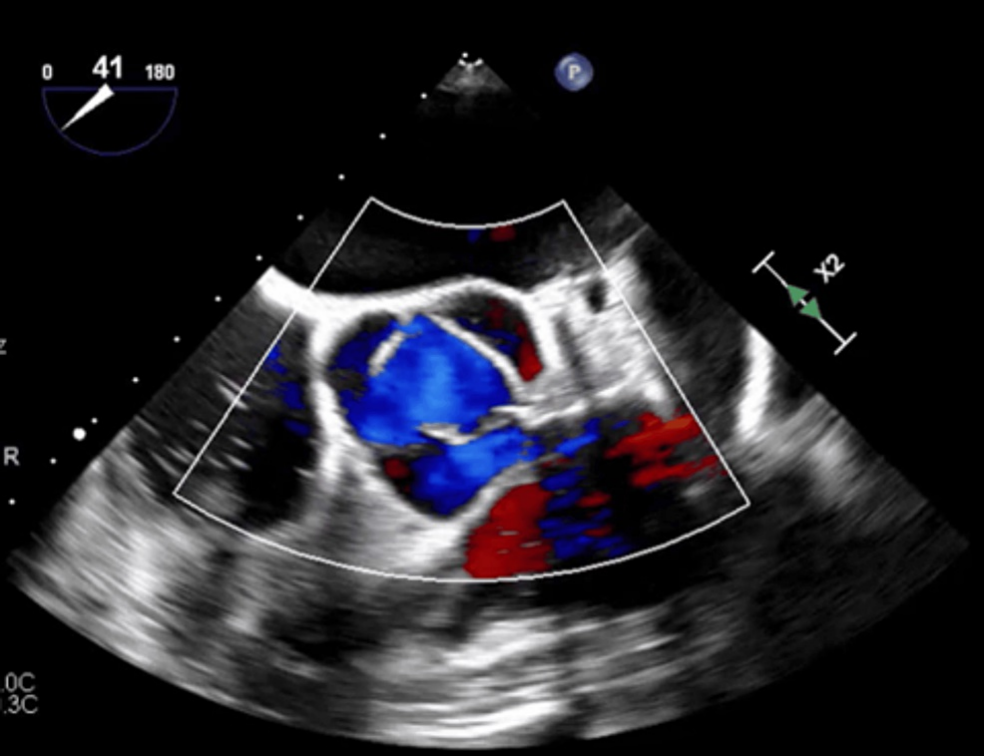
Post-procedural TEE showed a successfully ligated LAA with a very small residual scallop-shaped LAA with laminal flow. TEE: transesophageal echocardiogram; LAA: left atrial appendage

Since the patient was outside the tissue plasminogen activator (tPA) window, she was started on aspirin and clopidogrel for 21 days and switched to aspirin alone thereafter per neurology recommendation. Given her recent VAT left atrial appendage clipping, cardiothoracic surgery was consulted. They recommended no further surgical management as the patient had an unremarkable preprocedural transesophageal echocardiogram (TEE), and the post-procedural TEE showed successful ligation of the LAA with a very small residual scallop-shaped LAA with laminar flow (Figure 3). Cardiology also advised against further invasive testing, including a repeat TEE, considering there would be no change in management on account of the absolute contraindication to anticoagulation. The patient showed significant improvement in left-sided weakness, with a power of 5/5 in the upper extremity and 4/5 in the lower extremity upon discharge to a subacute rehab facility.

## Discussion

The LAA is the tubular outpouching of the left atrium, separated by an oval orifice that contains muscular trabeculae that facilitate the formation of thrombi in nonvalvular AF. This is where up to 90% of thrombi-causing ischemic strokes are thought to arise [7]. Over the past few decades, medical experts have attempted novel approaches to exclude this part of the atrium to prevent thrombus formation. Some of these approaches involve the occlusion of the LAA using endocardial devices, epicardial devices, including the AtriClip device, or a combination of both [9]. Endocardial devices are in contact with the circulation and require further anticoagulation for at least 45 days, by which time the device is expected to have endothelialized. This option, therefore, is not ideal for people with absolute contraindications to anticoagulation [10].

The epicardial approach, such as in the Atriclip procedure, provides a significant alternative, as no post-procedure anticoagulation is recommended if the LAA is successfully excluded [10]. Earlier multicenter trials found that LAA occlusion with AtriClip is safe and efficacious; those studies were limited by the small sample size and short follow-up period [9]. The post-procedure TEE and the interval transthoracic echocardiogram for our patient showed no intramural thrombus and a successfully ligated LAA. Hence, it remains unclear why our patient developed a cardioembolic stroke shortly after her procedure. A prospective cohort study assessing the long-term efficacy of AtriClip revealed a relative risk reduction in ischemic stroke by 87.5%, with two patients developing cardioembolic stroke [11].

Studies in the past have also found atrial mural thrombi in patients who developed cardioembolic events after surgical epicardial excision of LAA [11]. This is thought to be due to incomplete occlusion of the LAA due to a residual stump or a remnant distal part of the LAA with persistent blood flow [11]. However, these problems were not identified in our patient and also have not been identified in recent studies involving AtriClip [11].

As the evidence suggests, despite significantly reducing the incidence of stroke with AtriClip, there remains a residual risk for cerebrovascular events, which cannot be discounted due to the impact on morbidity and mortality. A multicenter cohort analysis of thoracoscopic LAA clipping reported a low incidence of cerebrovascular events of 0.5/100 person-years in the patients. However, the patients recruited received anticoagulation during the follow-up period [12]. Similar incidence rates of stroke were reported in large single-center studies, but no associated intra-atrial thrombi were found in persons who developed a stroke [13].

To the best of our knowledge, there are no evidence-based guidelines on the management of cardio-embolic stroke following an AtriClip procedure in patients who have an absolute contraindication to anticoagulation, as seen in our patient. Neurology was consulted and recommended three weeks of double antiplatelet therapy (DAPT), then continuation of aspirin indefinitely. 

## Conclusions

In our case, we did not find any apparent cause of the cardioembolic stroke. However, the patient’s history of paroxysmal AF, which is recurrent, makes it plausible that she might have had an episode that spontaneously cardioverted to normal sinus rhythm prior to presentation. Despite 90% of mural thrombi being found in the LAA in persons with nonvalvular AF, there remains another 10% inherent risk of ischemic stroke in these patients that is unrelated to the LAA thrombus and has a significant impact on health outcomes. Given that our patient had complete LAA occlusion with AtriClip, it is worth noting that this procedure did not completely exclude her risk of cardioembolic stroke, even though she was found to be in normal sinus rhythm.

There is, therefore, a need for a larger multicenter study to elucidate the frequency of post-procedural stroke and to evaluate the predisposition to stroke development post-AtriClip procedure, which would help guide further preventative actions.
